# Kaposi's Sarcoma-Associated Herpesvirus ORF6 Gene Is Essential in Viral Lytic Replication

**DOI:** 10.1371/journal.pone.0099542

**Published:** 2014-06-09

**Authors:** Can Peng, Jungang Chen, Wei Tang, Chunlan Liu, Xulin Chen

**Affiliations:** State Key Laboratory of Virology, Wuhan Institute of Virology, Chinese Academy of Sciences, Wuhan, Hubei, P.R. China; University of Southern California Keck School of Medicine, United States of America

## Abstract

Kaposi's sarcoma associated herpesvirus (KSHV) is associated with Kaposis's sarcoma (KS), primary effusion lymphoma and multicentric Castleman's disease. KSHV encodes at least 8 open reading frames (ORFs) that play important roles in its lytic DNA replication. Among which, ORF6 of KSHV encodes an ssDNA binding protein that has been proved to participate in origin-dependent DNA replication in transient assays. To define further the function of ORF6 in the virus life cycle, we constructed a recombinant virus genome with a large deletion within the ORF6 locus by using a bacterial artificial chromosome (BAC) system. Stable 293T cells carrying the BAC36 (wild type) and BACΔ6 genomes were generated. When monolayers of 293T-BAC36 and 293T-BACΔ6 cells were induced with 12-O-tetradecanoylphorbol-13-acetate (TPA) and sodium butyrate, infectious virus was detected from the 293T-BAC36 cell supernatants only and not from the 293T- BACΔ6 cell supernatants. DNA synthesis was defective in 293T-BACΔ6 cells. Expression of ORF6 in *trans* in BACΔ6-containing cells was able to rescue both defects. Our results provide genetic evidence that ORF6 is essential for KSHV lytic replication. The stable 293T cells carrying the BAC36 and BACΔ6 genomes could be used as tools to investigate the detailed functions of ORF6 in the lytic replication of KSHV.

## Introduction

Kaposi's sarcoma associated herpesvirus (KSHV), also known as human herpesvirus 8 (HHV-8), is a member of the gamma-2 herpesvirus family [1]. It is associated with Kaposi's sarcoma (KS), primary effusion lymphoma and multicentric Castleman's disease [2], [3]. Like other herpesvirus, the life cycle of KSHV consists of latent and lytic replication phases [4]. During latency, only a few viral genes were expressed [5] and there is no infectious virus production. KSHV genomes maintain as circular double-stranded DNA molecules (episomes) tethered to the host chromosome via LANA and are replicated in synchrony with host cells that depend on host cellular DNA polymerase and accessory factors. When latency is disrupted, KSHV switches to lytic phase [4], [6]. In the lytic phase, the virus expresses most or all of its genes and viral DNA is amplified via a rolling circle mechanism by utilizing its own DNA polymerase and other factors [7]. In KSHV-induced malignancies, a majority of the tumor cells are latently infected with KSHV and only a small percentage of cells (2%–5%) undergo lytic replication [8], [9]. Increasing evidence suggests that the small percentage of viral lytic replication plays great roles in viral pathogenicity. This spontaneous reactivation directly contributes to viral tumorigenesis through generation of virions for further spread infection and production of homologs of cellular cytokines which act in a paracrine manner for tumor progression [8].

Lytic DNA replication of KSHV initiates from two lytic origins (ori-Lyt-L and ori-Lyt-R) and requires many viral gene products. A transient cotransfection-replication assay has elucidated the set of *trans*-acting factors required for lytic DNA replication, these factors consist of homologues to the core replication proteins: DNA polymerase (Pol-8, encoded by ORF9), processivity factor (PF-8, encoded by ORF59), helicase (HEL, encoded by ORF44), primase (PRI, encoded by ORF56), primase-associated factor (PAF, encoded by ORF40/41), single-stranded DNA binding protein (SSB, encoded by ORF6) along with replication and transcription activator (RTA, encoded by ORF50) and virus specific origin binding protein (OBP, encoded by K8) [10]. The six core replication proteins could form large globular shaped pseudo-replication compartments which exclude cellular DNA even in the absence of a lytic cycle replication origin and any known origin binding protein. They could fully substitute for their Epstein-Barr virus (EBV) counterparts in the replication of EBV ori-Lyt [11].

ORF6 is defined as a delayed-early gene whose RNA is typically detected at 8–24 h postinduction [12], [13]. Its expression has been reported to be regulated by RTA which could bind to RBP-jk recognition site on ORF6 promoter via interaction with RBP-jk protein [14]. ORF6 encodes a single-stranded DNA binding protein with a predicted molecular mass of 126 kDa. It was identified from sequencing the KSHV genome [15] as having sequence similarity to EBV BALF2 protein and HSV-1 ICP8 protein, both of which have been characterized as single-stranded DNA binding proteins. In HSV-1, ICP8 has been shown by many groups to have multiple functions in viral DNA replication. It is implicated in the regulation of gene expression by repressing transcription from the parental genome [16] and stimulating late gene expression from progeny genomes [17]. Genetic studies have demonstrated that ICP8 is required for the localization of viral replication and cellular proteins to the replication compartments [18], [19]. ICP8 has been shown to interact either physically or functionally with several viral proteins to modulate their activities [20], [21], [22]. ICP8 has also been reported to interact with several cellular proteins known to be involved in recombination, including DNA-PKcs (DNA-dependent protein kinase, catalytic subunit), K86 and Rad50 and recruit these proteins to viral replication compartments [23]. A few reports have also demonstrated the essentiality of several γ-herpesvirus replication proteins in their lytic replication, these include the single-stranded DNA binding protein of EBV (BALF2) [24], ORF6 of murine γ-herpesvirus-68 (MHV-68) [25], [26], UL57 of human cytomegalovirus (HCMV) [27]. Based on these data and the similarities between lytic machinery among the herpesvirus subfamilies, it prompted us to test whether KSHV ORF6 is essential for viral lytic replication.

In order to facilitate the study of the function of ORF6 in the context of the KSHV genome during lytic replication, we constructed a ORF6-null virus by using the recombinant KSHV BAC36 as a initial template which allows the genetic manipulation of individual genes within the viral genome and the functional analysis of the resulting phenotype [28], [29], [30]. Our results demonstrated that the ORF6 null recombinant virus is deficient in infectious virus production and lytic DNA synthesis. However, BACΔ6 could be rescued upon introduction of ORF6 into BACΔ6 containing cells. Therefore, ORF6 is absolutely essential for the lytic DNA replication of KSHV.

## Materials and Methods

### Cells, Escherichia coli strains, antibodies and chemicals

Human embryonic kidney (HEK) 293T cells were maintained in Dulbecco's modified Eagle's medium supplemented with 10% fetal bovine serum and antibiotics. *E.coli* strain EL350 was a gift from Fanxiu Zhu (Florida State University). Mouse anti-RTA antibody was a gift from Ke Lan (Institute Pasteur in Shanghai, Chinese Academy of Sciences). 12-O-Tetradecanoylphorbol-13-acetate (TPA), sodium butyrate, and polybrene were purchased from Sigma. Hygromycin was purchased from Amresco.

### Plasmids

BAC36, which contains the entire KSHV genome, was kindly provided by Shou-Jiang Gao (University of Southern California). PCR fragments of full-length (nt 3210 to nt 6611, according to the HHV-8 genomic sequence, GeneBank: U75698) was cloned in pFLAG-CMV2 (Sigma) to construct N-terminal Flag fusion proteins. pCR3.1-ORF50 and pOri-A, which contain the ORF50 coding region and lytic replication origin of KSHV respectively, were kindly provided by Yan Yuan (University of Pennsylvania). pTOPO10-Kan/SacB were kindly provided by Fanxiu Zhu (Florida State University).

### Construction of mutant BACΔ6

Mutagenesis of BAC36 was performed using a recombineering system (http://recombineering.ncifcrf.gov). 100 ng purified BAC36 DNA was first introduced into EL350 by electroporation. The EL350 strain contains a defective λ prophage with recombination proteins Exo, beta and gam controlled by the temperature-sensitive repressor cI85. When the culture was incubated in 42°C for 15 min, recombinant functions could be supplied transiently [31]. To construct an ORF6 deletion mutant, we replaced the ORF6 coding sequence of BAC36 with kanamycin (Kan)/SacB cassette by homologous recombinant. We kept the last 1010 bp of ORF6 in case of not interrupting the expression of ORF7 since its stop codon is just 18 bp upstream of the start codon of ORF7.The Kan/SacB cassette, which contains the kanamycin resistance gene and SacB, was amplified from plasmids pTOPO10-Kan/SacB with primers ORF6-Kan/SacB-F and ORF6-Kan/SacB-R. Each primer contains 21 nucleotides (nt) homologous to the antibiotic resistance Kan/SacB cassette at its 3′ end and 50 nt next to the start or nucleotides 5600 of ORF6 at the 5′ end. The PCR product was treated with *Dp*nI (New England Biolabs) at 37°C for 2 hrs to remove the plasmid template, and then purified with TIANGEN gel extraction kit (TIANGEN). The 200 ng purified DNA was electroporated into BAC36-containing EL350 which incubated at 32°C until the OD_600_ reached 0.6, and induced at 42°C for 15 min. Electroporation was performed with a Bio-Rad GenePulser Electroporator under the following condition: 1.75 kV, 25 µF with pulse controller set at 200 ohms in a 0.1 cm cuvette. The recombinant clones were selected at 32°C on LB plates with 12.5 µg chloramphenicol and 50 µg of kanamycin per ml and analyzed by PCR and Southern blot. The mutant BAC36 was designated BACΔ6.

### Confirmation of the ORF6 deletion in BACΔ6 DNA

The initial confirmation of the colonies on LB agar plates containing chloramphenicol and kanamycin was performed by colony PCR using two pairs of primers. Primers A and B recognize sequence outside ORF6 sequence while primers C and D amplify a ∼1.2-kb ORF6 fragment within the deleted region. Restriction enzyme digestion was carried out to analyze BAC36 and BACΔ6 genomes. DNAs were isolated from bacteria harboring BAC36 and BACΔ6 by using a NucleoBond Xtra Midi kit (Macherey-nagel, German). BAC DNAs were digested with *Bg*lII and run on 0.8% agarose gels. Samples were prepared in triplicate. One was visualized by ethidium bromide staining and the other two were denatured, transferred to Nylon+ membrane (GE health). Prehybridization was performed at 68°C for 30 min in DIG hybridization buffer (Roche). The two DNA blots were hybridized to two DIG-labeled probes separately that prepared through incorporating DIG-11-dUTP (Roche) into fragments from ORF6 and Kana/SacB cassette by PCR amplification in the DIG hybridization buffer at 48°C for 6 h. Blots were washed twice for 15 min with 2×SSC (1×SSC is 0.15 M NaCl plus 0.015 M sodium citrate) - 0.1% sodium dodecyl sulfate (SDS) and twice for 15 min with 0.5×SSC - 0.1% sodium dodecyl sulfate at 68°C. Blots were blocked in 1% blocking solution (Roche) for 30 min and then incubated with diluted AP-labeled anti-DIG antibody (Roche) for 30 min. The CDP-star chemiluminescence system (Roche) was used to detect the DIG antibody-antigen complexes.

### Establishment of stable BAC36 and BACΔ6-containing 293T cell lines

293T cells were transfected with purified BAC36 or BACΔ6 DNAs using Lipofectamine 2000 (Invitrogen) according to the manufacture's instructions. Two days after transfection, the cells were examined by fluorescence microscopy and then subcultured with fresh medium containing 400 µg/ml hygromycin. Fourteen days after transfection, the colonies were visible. The colonies were dislodged and seeded into a new culture flask. The stable cell line harboring BAC36 and BACΔ6 were cultured with 250 µg/ml hygromycin.

### RT-PCR

Total RNA was prepared, treated with DNase I (Promega) and converted to cDNA by a first-strand synthesis system (Promega) and random hexamers. The cDNA samples were used in each reaction for the amplification of viral transcripts using transcript-specific primers (listed in table 1). β-actin was used as the normalization control for input RNA. RT-PCR products were run on 1.5% agarose gel and analyzed. Amplification of DNase I-treated RNA without RT reaction confirmed the absence of any contaminating DNA in the cDNA samples.

**Table:** **Table1.** Primers used for PCR

Primer	sequence
ORF6-Kan/SacB-F	CAAGCCATTATACACACGGGTTTTTTGTTGTCTTGGCCAATCGTGTCTCCGAATTCGCATGCGACGTCCACATATAC
ORF6-Kan/SacB-R	CTTGCCTTTACATAATTACATAACTCCGTTAGGTGTTCCCCCGAGACACCCTACCGCACAGATGCGTAAGG
Primers A	GGGAAAGCGACAGAAGG
Primers B	CATAGACCGCCGCCAGTT
primers C	CGCTAAAGGGACCACAAACC
primers D	AGGTGCTGGAGGGTGTAAGA
ORF6-F	CATCAAATCCGTCCATCA
ORF6-R	TGCAAACATCCCTCCTAT
ORF7-F	ACTGTGGGGGTCTGTCATCT
ORF7-R	GGTTGAAGTTGGGCTCTATC
ORF50-F	GCCCTCTGCCTTTTGGTT
ORF50-R	GATGATGCTGACGGTGTC
ORF65-F	ATATGTCGCAGGCCGAATAC
ORF65-R	CCACCCATCCTCCTCAGATA
ORF73-F	GGAATGCGCCTGAGGTCGGGACGGA
ORF73-R	GGCAGCCCGGATGTGAACACT
β-Actin-F	CTCCATCCTGGCCTCGCTGT
β-Actin-R	GCTGTCACC TTCACCGTTCC

### Western blot analysis

Cells were washed with 1×PBS and lysed with RIPA lysis buffer (50 mM Tris-HCl, pH 7.4, 150 mM NaCl, 0.1% SDS, 1% Triton X-100, 1% deoxycholate, 1 mM, sodium orthovanadate [Na3VO4], 20 mM sodium pyrophosphate, 100 mM sodium fluoride, 10% glycerol, 1 mM EDTA, 5 µg/ml of aprotinin, 5 µg/ml of leupeptin, 5 mM benzamidine, and 1 mM phenylmethylsulfonyl fluoride). The cell lysates were homogenized and centrifuged at 13,000 rpm for 20 min at 4°C. Fifty micrograms of whole-cell extracts were resolved by SDS-polyacrylamide gel electrophoresis and transferred to PVDF membranes. The membranes were blocked in 5% nonfat dry milk solution in TBST buffer (10 mM Tris-HCl, PH 7.2, 150 mM NaCl) plus 0.1% Tween 20 and then incubated with diluted primary antibodies for 2 h at room temperature. Anti-rabbit or anti-mouse immunoglobulin G antibodies conjugated to horseradish peroxidase (Thermo) were used as the secondary antibodies. An enhanced chemiluminescence system (Pierce) was used for detection of antibody-antigen complexes.

### Infection of cells with harvested supernatants

293T cells plated in 24-well were incubated with supernatants plus polybrene (8 µg/ml) and spun at 2200 rpm for 1 h at room temperature, and then were incubated at 37°C for another 2 hours. The inocula were removed and replaced with fresh media with 5% FBS. The following day, the media were replaced with fresh media containing 1% FBS. Green fluorescent protein (GFP) expression was used to monitor the infection 2 days after infection.

### Quantitation of genomic and viral DNAs

Total DNAs prepared from cells were extracted with a DNA purification kit (Beyotime Biotechnology, Hangzhou, China). The monolayer of 293T-BAC36 and 293T- BACΔ6 cells at 0, 48 and 72 h postinduction were trypsinized, washed and resuspended in 200 µl of 1×PBS. Total DNA was prepared according to the manufacturer's instructions. For DNA extraction from virus stock, virions released into the extracellular medium 4 days post-induction were purified and concentrated from the medium supernatant as described earlier [29]. Virus stock (180 µl) were prepared with 10 µl of DNase I (Fermentas) at 37°C for 60 min to ensure removal of any contaminating DNA outside viral particles. DNAs were prepared using a NucleoSpin Blood Kit (Macherey-nagel, German). Copy number of KSHV genomic DNA in viral stocks and cells were estimated by real-time DNA PCR containing primers for detection the KSHV ORF73 gene [32]. Viral genomic DNA copy numbers obtained from viral stocks were expressed as copy number/ml of the medium supernatant, while the intracellular viral copy numbers were normalized to that of GAPDH.

### Transient DNA replication assay

To assay ori-Lyt-dependent DNA replication, 293T-BAC36 and 293T-BACΔ6 cells were transfected with the plasmids pOri-A (2 µg), pCR3.1-ORF50 (1 µg) and pFlag-ORF6 (1 µg) as indicated using lipofectamine 2000. Plasmids Flag-cmv2 was added to maintain the same amount of total transfected DNA. At 72 h post-transfection, extrachromosomal DNAs were prepared from cells by using the Hirt DNA extraction method. Briefly, cells were lysed in 700 µl of lysis buffer (10 mM Tris-HCl [pH 7.4], 10 mM EDTA, 0.6% sodium dodecyl sulfate and 50 ug/ml RNase A). Chromosomal DNA was precipitated at 4°C overnight by adding 5 M NaCl to a final concentration of 0.85 M. The supernatant containing extrachromosomal DNA was subjected to phenol-chloroform extraction, followed by ethanol precipitation. Then 15 µg Hirt DNA was digested with *Eco*RI/*Ps*tI/*Dp*nI (New England Biolabs). The DNAs were separated by electrophoresis on a 0.8% agarose gel transferred onto a Nylon+ membrane and hybridized with DIG-labeled pOri-A probe.

### Complementation of 293T-BACΔ6 cells with lentiviruses expressing ORF6

293T-BACΔ6 cells were incubated with lentiviruses expressing ORF6 plus polybrene added to a final concentration of 5 µg/ml for 5 h and then replaced with fresh medium. Forty eight hours postinfection, cells were induced with 25 ng/ml TPA and 1.5 mM sodium butyrate. Cells and supernatants were harvested at the indicated time points, intracellular and extracellular viral DNAs were extracted as described above.

## Results

### Generation of ORF6-null recombinant KSHV

**Figure 1:**
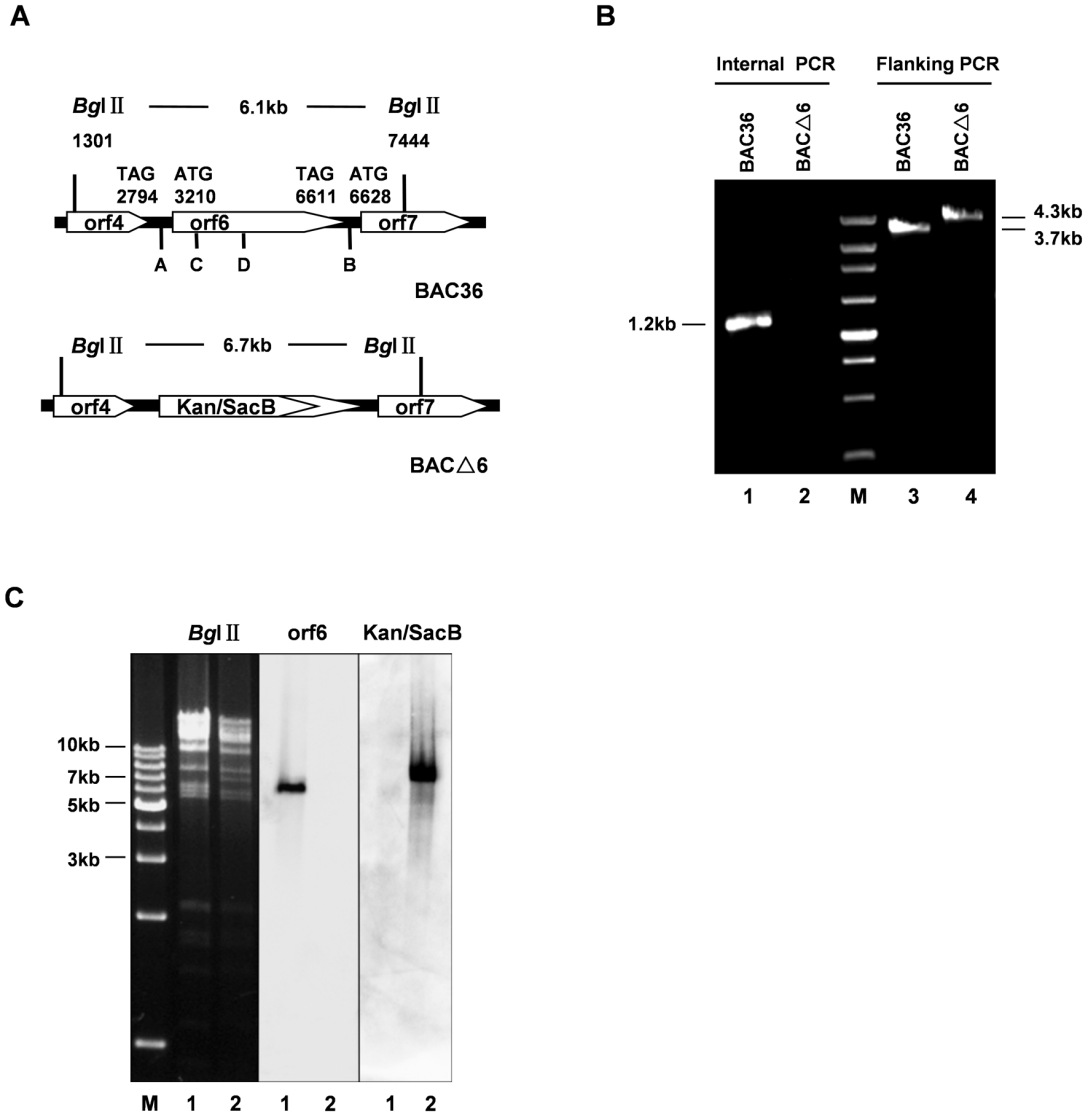
Construction and analysis of ORF6-null KSHV genome. (**A**) Schematic diagrams of the structures of ORF6 and its neighboring ORFs in the wild-type and mutant BACs. (**B**) Confirmation of KSHV ORF6 replacement with Kan/SacB cassette by PCR amplification. The BAC36 (lane 1 and 3) and BACΔ6 (lane 2 and 4) were amplified with flanking PCR primers A and B and internal PCR primers C and D. (**C**) Confirmation of BAC36 and BACΔ6 genome with *Bg*lII digestion and Southern blot analysis. BAC36 (lane 1) and BACΔ6 (lane 2) DNAs were digested with *Bg*lII. The digested DNAs were electrophoresed, blotted, and hybridized with Dig-labeled ORF6 or Kan/SacB DNA probes, respectively.

Cloning of the full KSHV genome as a bacterial artificial chromosome (BAC), BAC36, has greatly facilitated the genetic manipulation of the KSHV genome in *E.coli* [28]. Infectious virus can be reconstituted by transfection of BAC36 into 293T cells. The BAC36 contains GFP and hygromycin resistance genes which allow easy detection of eukaryotic cells carrying KSHV genomes and establishment of stable cell lines. To investigate the roles of ORF6 in the viral lytic life cycle, we constructed the BACΔ6 KSHV genome by replacing a 2391 bp ORF6 region in the BAC36 KSHV genome with a Kan/SacB cassette using recombineering technology (recombination-mediated genetic engeneering). Briefly, a bacterial double selection cassette, Kan/SacB, flanked by sequence homologous to the first 50 nt prior to the initiation codon of ORF6 and the 50 nt beginning at nucleotide position 2392 of ORF6 at two ends was synthesized by PCR. We kept the last 1010 bp of ORF6 to avoid interrupting the expression of ORF7 since its start codon is just 18 bp downstream of the stop codon of ORF6. Then the purified PCR product was transformed into *E.coli* EL350 cells carrying BAC36. Induction of recombination activity in the EL350 cells at 42°C resulted in the replacement of major portion of the ORF6 by the Kan/SacB cassette. Transformants were selected by kanamycin resistance (Kan^+^). Colonies resistant to kanamycin were first screened by PCR for detection of special fragments using flanking primers (A and B), which recognize sequence outside the ORF6. Flanking primers amplified a 4.3-kb product from the BACΔ6 genome and a 3.7-kb product from the BAC36 genome (Fig. 1B, lane 3 and 4). Positive colonies were further screened by another round of PCR internal primers (C and D) which amplify a region inside the deleted fragment. The internal primers did not amplify any product from BACΔ6 DNA, while amplified a fragment of 1226-bp from BAC36 DNA (Fig. 1B, lane 1 and 2). BAC36 and BACΔ6 DNAs were isolated, digested with restriction enzymes, and analyzed on 0.8% agarose gels. Digestion of the wild-type BAC36 DNA with *Bg*lIIgenerated a 6.1-kb fragment at the ORF6 locus. Replacement of the ORF6 2391-bp region with the Kan/SacB cassette shifted the fragment size to 6.7 kb. The southern blot hybridizations were performed to confirm further that the altered digestion pattern of the BACΔ6 mutant was the result of the expected recombination. The restriction-digested DNAs were transferred onto nylon membranes and probed with DIG-labeled ORF6 sequence missing in BACΔ6 and sequence within the Kan/SacB DNA, respectively. When probed with ORF6 sequence, a 6.1 kb fragment was only detected in wild-type BAC36 and not in the BAC mutant (Fig. 1C, lane 1 and 2). When the same blot was probed with Kan/SacB DNA, the 6.7 kb band was only seen in BACΔ6 (Fig. 1C). These results confirmed that the major ORF6 coding sequence was successfully replaced with a Kan/SacB cassette.

### Generation of stable 293T cells carrying BAC36 and BACΔ6 genomes

**Figure 2:**
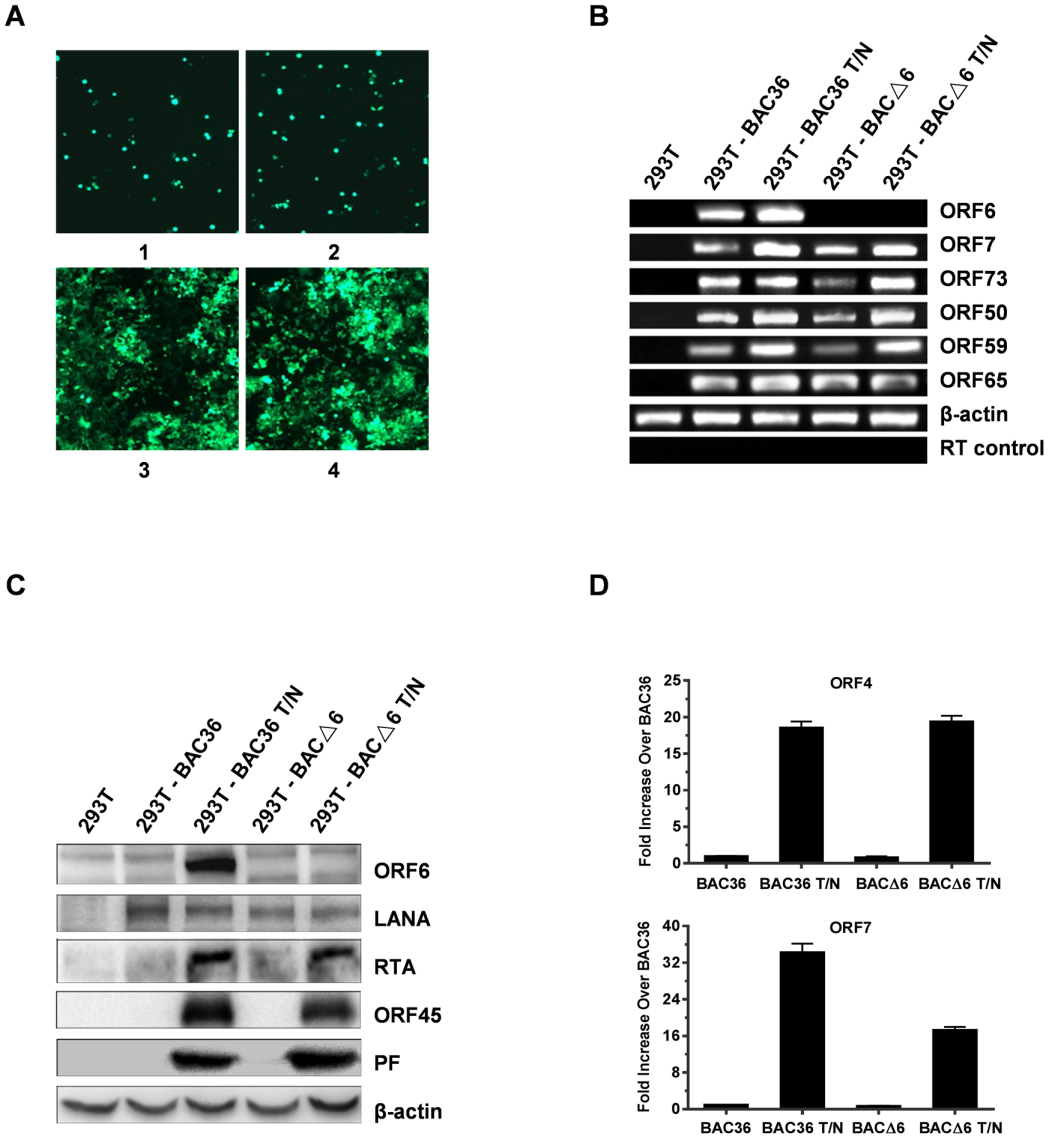
Generation of stable 293T cells carrying BAC36 and BACΔ6 genomes. (**A**) Transfection of 293T cells with BAC36 and BACΔ6 DNAs. Cells were transfected with BAC36 (1 and 3) and BACΔ6 (2 and 4) with lipofectamine 2000. GFP expression levels were monitored by fluorescent microscopy 2 days post-transfection (1 and 2). Then, the transfected cells were split and selected with hygromycin. Hygromycin-resistant clones of 293T-BAC36 and 293T-BACΔ6 cells were established (3 and 4). (**B**) RT-PCR confirmation of the absence of ORF6 in 293T-BACΔ6 cells. Total RNA from uninduced or TPA/NaB induced 293T-BAC36 cells and 293T-BACΔ6 cells were used to prepare cDNAs. The RT-PCR generated products were analyzed on a 1.5% agarose gel. Shown are the KSHV ORFs 6, 7, 73, 50, 59 and 65, respectively. β-actin was used as the normalization control for input RNA. Absence of contaminating DNA in the samples was tested by reverse transcriptase-negative control reactions (RT control). (**C**) Western blot confirmation of the absence of ORF6 in 293T-BACΔ6 cells. The 293T-BAC36 cells and 293T-BACΔ6 cells were induced with TPA/NaB for 2 days. Whole cell lysates were immunoblotted with antibodies against ORF6, LANA, RTA, ORF45 and ORF59. The same blots were probed with anti-β-actin antibody to ensure equal loading of all samples. (**D**) Real-time PCR analysis of the mRNA levels of ORF4 and ORF7 from 293T-BACΔ6 cells induced with TPA/NaB. cDNAs were prepared as described previously and analyzed by real-time PCR using primers specific for ORF4 and ORF7 transcripts. β-actin was used as an internal standard. The data are shown as the fold increase compared to the untreated 293T-BAC36 cells.

To reconstitute recombinant viruses, BAC36 and BACΔ6 DNAs were transfected into 293T cells, which exhibit high transfection efficiency and permissiveness for KSHV lytic cycle [33]. The presence of GFP makers and hygromycin resistance in the BAC36 genomes enabled us to monitor the transfection efficiencies by GFP detection and to get single GFP-expressing colonies by hygromycin selection. After three weeks selection, homologous population of cells harboring BAC36 and BACΔ6 were obtained (Fig. 2A). Stably transfected cells were induced with TPA and sodium butyrate. Total RNAs were isolated from induced and uninduced cells, treated with DNase I, and analyzed by RT-PCR for KSHV transcripts. As expected, no ORF6 transcripts were detected in 293T-BACΔ6 cells (Fig. 2B), whereas they could be easily detected in 293T-BAC36 cells. Other viral transcripts, latent ORF73, immediate-early ORF50, early ORF59, late ORF65, were all detected in both type of cells (Fig. 2B). In addition, whole cell lysates from induced and uninduced cells were analyzed for expression of ORF6 by Western blot analysis. ORF6 was not detected in 293T- BACΔ6 cells, whereas it could be detected easily in 293T- BAC36 cells (Fig. 2C). Moreover, the Western blot analyses detected no significant difference between BAC36 and BACΔ6 in expression of LANA, ORF45, RTA and PF. To exclude the possibility that the replacement of the large part of the ORF6 sequence with Kan/SacB may cause defects on the expression of neighboring genes ORF4 and ORF7, the transcription levels of ORF4 and ORF7 from both cells were examined with real-time PCR. As shown in figure 2D, no significant difference was found in the expression of ORF4 between the induced 293T-BAC36 and 293T-BAC△6 cells. An 18.6-fold increase in the expression of ORF4 was observed in 293T-BAC36 cells treated with TPA-butyrate comparing with that in 293T-BAC36 cells without induction, while a 19.4-fold increase was detected in 293T-BAC△6 cells treated with treated with TPA-butyrate at 48 h postinduction. The expression of ORF7 increased up to 34.2-fold in the induced 293T-BAC36 cells over that in uninduced 293T-BAC36. A 17.3-fold increase was observed in the induced 293T-BAC△6 cells, which suggested that the transcription of ORF7 was still active although the level was lower than that in the induced 293T-BAC36 cells. Thus, replacement of ORF6 did not impair the transcription of ORF4 and ORF7. These results confirmed the deficiency of ORF6 transcription and expression from BACΔ6 genome.

### 293T-BACΔ6 cells failed to produce virus unless trans-complemented by ORF6

**Figure 3:**
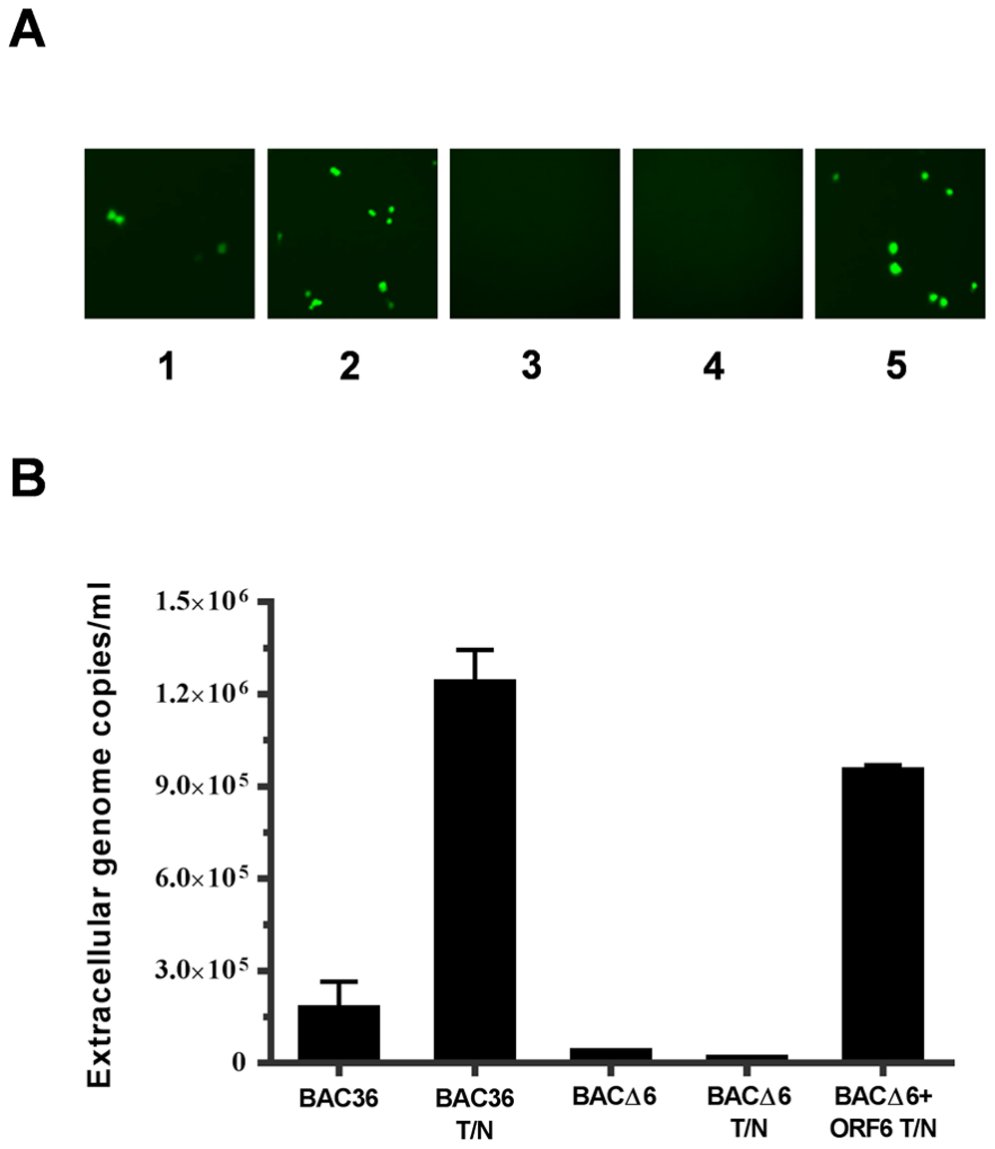
Extracellular progeny virion production of 293T-BAC36, 293T-BACΔ6 and 293T-BACΔ6 complemented with ORF6. (**A**) Infectivities of supernatants from 293T-BAC36, 293T-BACΔ6 and 293T-BACΔ6 cells complemented with ORF6 in the absence or presence of TPA/NaB. 293T cells were innoculated with supernatants from uninduced 293T-BAC36 (1) and 293T-BACΔ6 (3), induced 293T-BAC36 (2), 293T-BACΔ6 (4) and 293T-BACΔ6 complemented with ORF6 (5). GFP expression was examined under a fluorescence microscope at 2 days postinfection. (**B**) Quantitation of viral genomic DNA in supernatants from 293T-BAC36, 293T-BACΔ6 and 293T-BACΔ6 complemented with ORF6 in the absence or presence of TPA/NaB. At 4 days post-induction, viruses in the supernatants were harvested and concentrated 100-fold. Viral stocks were treated with DNase I for 1 h at 37°C, and viral DNAs were extracted. Viral DNAs were analyzed by a real-time PCR assay using primers to LANA. Copy numbers were normalized and are expressed as copy number per milliliter of supernatant.

To determine the effect of ORF6 deletion on infectious virus production, supernatants at 96 h from uninduced and induced 293T-BAC36 and 293T-BACΔ6 cells were harvested and used to infect 293T cells. Infected cells were examined for GFP expression 2 days postinfection. As shown in Fig. 3A, the induced 293T-BAC36 cells (panel 2) were capable of producing infectious progeny virus, as demonstrated by the appearance of green cells upon inoculation of fresh 293T cells. The uninduced 293T-BAC36 cells could produce a much smaller number of progeny virions, which could represent cells spontaneously entering the lytic cycle (panel 1). The inoculation of fresh 293T cells with supernatants from uninduced (panel 3) or induced (panel 4) 293T-BACΔ6 cells could not produce green cells, indicating there is no production of infectious virions. However, when ORF6 was introduced into the induced 293T-BACΔ6 cells using lentivirus expression system, infectious viruses were produced, as revealed by the appearance of green cells upon inoculation of fresh 293T cells (panel 5). To further confirm the above results, the infected cells were subcultured in 60-mm dishes with fresh media containing 250 µg/ml hygromycin. After 2 weeks of selection, colonies were formed and analyzed. There was no ORF6 expression from cells infected with supernatants collected from 293T-BACΔ6 cells trans-complemented with ORF6 (data not shown). To determine the amounts of virus production, the supernatants were concentrated and treated with DNase I to remove any contaminating DNA outside viral particles, the encapsidated viral DNAs in the supernatants were quantified by real-time PCR analysis. For the induced wild-type BAC36, the viral copy number was about 1.3×10^6^/ml. The capability of virus production from the induced 293T-BACΔ6 cells trans-complemented with ORF6 was comparable to that from wild-type BAC36 cells (Fig. 3B). These results demonstrated that ORF6-null virus could produce infectious virus when ORF6 is supplied in *trans* and that the mutation within the KSHV genome only affects the ORF6 locus.

### ORF6 is essential for KSHV lytic DNA replication

**Figure 4:**
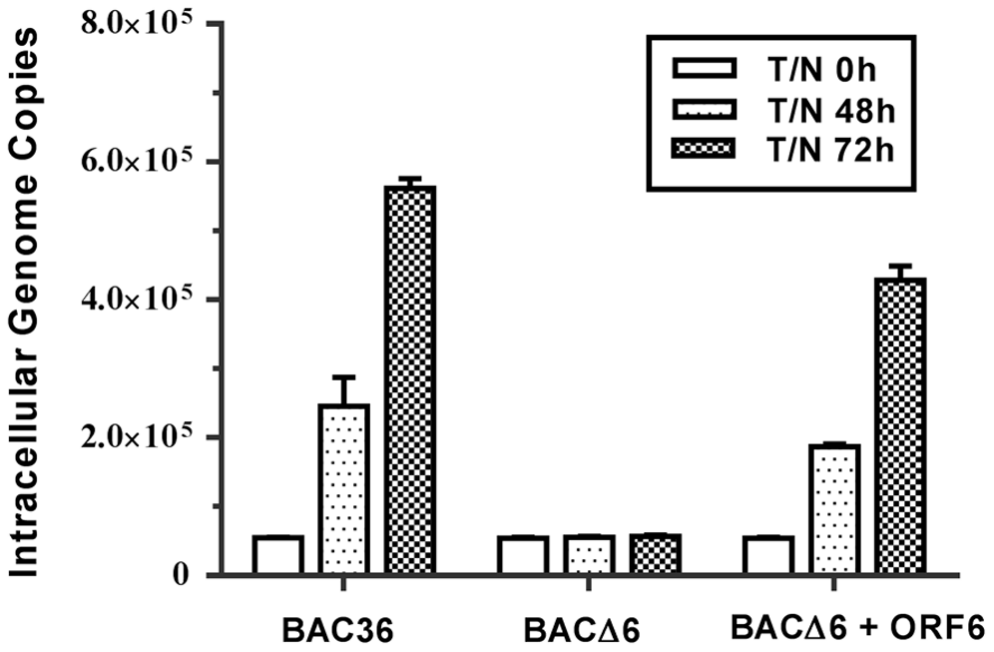
Lytic DNA replication of 293T-BAC36 and 293T-BACΔ6 cells. 293T cells harboring BAC36, BACΔ6 and BACΔ6 infected with lentiviruses expressing ORF6 were collected at different time points postinduction (0, 48 and 72 h), and total intracellular DNA were extracted. Intracellular viral DNAs were measured by a real-time PCR with primers directed to the ORF73 gene. The viral genome copies were normalized to 20,000 copies of GAPDH.

**Figure 5:**
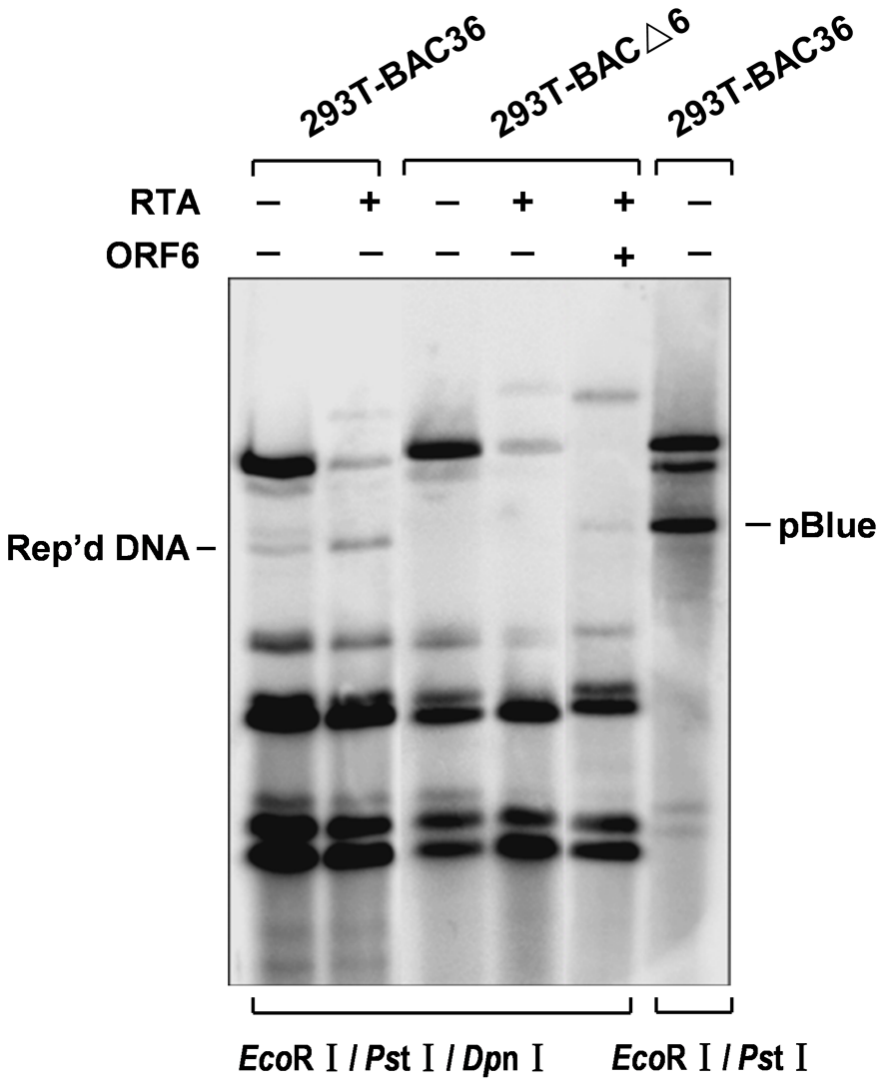
Ori-Lyt dependent lytic replication of 293T-BAC36 and 293T-BACΔ6 cells. 293T-BAC36, 293T-BACΔ6 cells were transfected with ori-Lyt plasmids (pOri-A) plus others as indicated (PCR3.1-ORF50 and FLAG-ORF6). KSHV lytic replication was induced by the expression of ORF50. Total DNAs were isolated from the transfected cells. Replicated DNAs were distinguished from input DNAs by *Dp*nI digestion and detected by southern blotting with Dig-labeled pOri-A probe.

ORF6 was found to function as a component in the viral replication complex in a transient transfection assay, indicating that ORF6 may play role in KSHV lytic DNA replication [10], [11]. This prompted us to use the ORF6 null recombinant virus to examine the role of ORF6 in viral lytic DNA synthesis in the context of the whole virus. Intracellular viral DNAs in stable 293T-BAC36 and 293T-BACΔ6 monolayer cells prior to and after TPA-butyrate induction were isolated and estimated using real-time PCR. As shown in Fig. 4, our results indicated 4.5-and 10.2-fold increases in intracellular DNA in wild-type virus upon induction for 48 h and 72 h while no increases was detected in BACΔ6 mutant virus. Of note, we observed 3.4-and 7.8-fold increase in genome copy numbers with ectopic expression of ORF6 in 293T-BACΔ6 cells. Next, to understand whether the viral DNA replication is ori-Lyt dependent, a plasmid carrying a KSHV ori-Lyt sequence (pOri-A) was tranfected into 293T-BAC36 and 293T-BACΔ6 cells, respectively. An RTA-expression vector was cotransfected for the induction of cells entry into lytic phase. Three days post transfection, Hirt DNAs were extracted and treated with *Eco*RI/*Ps*tI/*Dp*nI and analyzed by Southern blotting. As shown in Fig. 5, *Dp*nI-resistant products could be detected in 293T-BAC36 no matter whether cells were transfected with ORF50 or not. The replication product band could also be detected when ORF6 and RTA were cotransfected into 293T-BACΔ6 cells. These results confirm that in cells, ORF6 is essential for KSHV lytic replication.

## Discussion

KSHV ORF6 encodes a single-stranded DNA binding protein. It's a relatively abundant protein throughout the lytic cycle and packaged in the virions [34]. Its homologs, including HSV-1 ICP8, EBV BALF2 and VZV ORF29 [24], [35], [36], have been found in other herpesviruses and the requirement of these genes for viral lytic replication has been confirmed. ORF6 has 23% and 41% sequence identities to ICP8 and BALF2, respectively. A transient cotransfection-replication assay has elucidated the requirement of ORF6 for lytic DNA replication. But its contribution to lytic replication in the presence of the whole KSHV genome remains unknown. Therefore, the development of BAC36 system with deletion in ORF6 provides a fast and easy way to define the function of this gene.

In the present study, we genetically analyzed the importance of KSHV ORF6 for KSHV lytic growth by construction of an ORF6-null genome. Because the neighboring orf7 was only 18 bp away from orf6, the deletion of the entire ORF6 gene may influence the expression of the neighboring gene. We kept the last 1010 bp of ORF6 coding region. Besides, we measured the mRNA levels of ORF4 and ORF7 from both cells. No significant difference was found in the expression of ORF4 between 293T-BAC36 and 293T-BAC△6 cells postinduction. But the expression level of ORF7 in induced 293T-BAC△6 cells was only half of that in induced 293T-BAC36 cells even though an increase about 17.3-fold of ORF7 expression was detected in induced 293T-BAC△6 cells comparing with that in 293T-BAC36 cells without induction. It has been reported that CDV, an inhibitor of KSHV DNA replication, was used to dissect KSHV lytic gene expression into two components: genes expressed without DNA replication and those required it. ORF4 was characterized as a CDV-insensitive gene. ORF7 was defined as a CDV-sensitive gene whose expression decreased 50% at 48 h after induction when DNA replication was inhibited [37]. Considering that BAC△6 failed to replicate lytic DNA, the difference in the expression of ORF7 was in accordance with the pattern described previously. Hence, deletion of most region of ORF6 did not cause defects on expression of ORF4 and ORF7. Replacing a large portion of ORF6 gene rendered the KSHV genome from expressing SSB, producing infectious viruses and lytic DNA synthesis. These deficiencies could be rescued by infection with lentiviruses expressing ORF6. Our results demonstrated that ORF6 is essential in KSHV viral lytic replication. Construction of KSHV BAC36 by inserting a BAC cassette between ORF18 and ORF19 of a KSHV genome in the PEL cell line BCBL-1 allows the mutagenesis of individual gene in the complete genome and the functional analysis of the resulting phenotype. And it has been used successfully in many laboratories to analyze the function of several KSHV genes [29], [38], [39], [40]. It was reported that BAC36 contains a duplication of a 9-kb fragment of the long unique region, covering a part of ORF19, and the complete ORFs 18, 17, 16, K7, K6, and K5 [41]. So the duplicated region should be taken into consideration if mutagenesis or knock out of viral genes located within this region. Fortunately, ORF6 is not in this region. Southern blot and western blot analysis have confirmed the gene defect in BAC△6. It was also suggested that the 9-kb fragment in the TR region would lead to homologous recombination and the loss of the intact LUR region [42], [43]. *Wang* et al. in their article on functional analysis of K8 also mentioned that BAC-cloned KSHV genome, both wild type and mutants, become unstable in mammalian cells that have been cultured longer than two months [29]. Therefore, the freshly transfected cells were used for analysis and cells were cultured no more than 2 months in this study. And no significant change on GFP signal was observed during the time course. It appeared that the BAC36 system did not affect the functional analysis of ORF6.

Single-stranded DNA binding proteins of herpesvirus were reported to regulate gene expression. It is found that defects in HSV-1 ICP8 resulted in increased transcription of the ICP8, ICP5 and gC gene from parental genomes, implying its negative regulatory effect [44]. And analysis on a trans-dominant mutant of ICP8 indicated that ICP8 inhibits the expression of gC, UL47 and gD at the transcriptional level [17]. Either deletion or overexpression of VZV ORF29 would impair the expression of late gene gE [45]. ORF29 also enhanced the ability of ORF62 protein to transactivate the gI promoter in transfections [46]. In our study, we measured the mRNA level of ORF73 (latency), ORF50 (immediate early), ORF59 (early) and ORF65 (late). The abundancy of these genes in BACΔ6 cells was comparable to that in BAC36 cells. It is possible that KSHV ORF6 could also have regulatory effects, just like ICP8 and ORF29, on the expression of viral genes that haven't been detected. Further experiments need to be done to validate this hypothesis.

ORF6 is highly expressed during lytic phase and is believed to interact with multiple viral and cellular proteins. For instance, prereplication complex formed by the six core machinery proteins was recruited to ori-Lyt through K8 and RTA and together with several cellular proteins, such as topoisomerases I and II, MSH2/6, RecQL, and to initiate the lytic DNA replication in viral replication compartments. Interactions between SSB and K8, RTA and PF that did not rely on DNA have been detected [47]. Several other viral protein interactions with SSB by yeast two-hybrid and coimmunoprecipitation analysis were also identified [48]. However the significance of these interactions is still unclear. The ORF6-null recombinant virus will allow us to understand the functions of such interactions. Taken together, our findings in this study provide genetic evidence that ORF6 is essential for KSHV lytic replication. The stable 293T cells carrying BACΔ6 genome could be used as convenient tools to investigate the detailed functions of ORF6 in the lytic cycle of KSHV replication.
